# Reflections on two years after establishing an orthogeriatric unit: a focus group study of healthcare professionals’ expectations and experiences

**DOI:** 10.1186/s12913-017-2550-3

**Published:** 2017-08-25

**Authors:** C. Abrahamsen, B. Nørgaard, E. Draborg, D. Nielsen

**Affiliations:** 1 Department of Orthopaedic Surgery, Kolding Hospital, Sygehusvej 20, 6000 Kolding, Denmark; 2 Department of Public Health, University of Southern Denmark, J.B Winsløws Vej 9B, 5000 Odense, Denmark; 3 Centre for Global Health, University of Southern Denmark, Kløvervænget 6, 8th floor, 5000 Odense, Denmark

**Keywords:** Orthogeriatric care, Healthcare professional, Interprofessional collaboration, Focus group

## Abstract

**Background:**

For decades hospitals have been “vertically” organized, with the risk that specialization leads to fragmented and one-sided views of patient care and treatment that may cause poor communication and coordination of care and treatment. Two years after the introduction of an orthogeriatric unit for elderly patients admitted with fragility fractures, we studied the involved healthcare professionals’ perspectives and experiences with working in an interprofessional organization.

**Methods:**

We performed four focus groups interviews with 19 healthcare workers representing different professions. The interviews were analysed using systematic text condensation (STC).

**Results:**

Three themes were identified: 1) A patient-centred approach, 2) An opportunity for professional growth and 3) The benefits of interprofessional collaboration. The interviewees emphasized in particular the systematic and frequent face-to-face communication enabled by the interprofessional team meetings as essential to their feeling of enhanced collegial solidarity. All groups expressed their respect for other groups’ competences and their vital contributions to good orthogeriatric care. However, collaboration was challenged by the groups’ divergent views of the patients and of the relevance of the information given in the weekly meetings. Heavy workloads were also mentioned. The opportunity for professional growth was also felt to be imperilled by some professionals.

**Conclusions:**

All participants indicated their view that the orthogeriatric organization had improved the quality of care and treatment. Furthermore, good communication, mutual respect for other professional competences and shared goals were found to have enhanced interprofessional collaboration and improved the sense of having a shared mission. However, differences in approaches and expectations continued to challenge the orthogeriatric model after 2 years. Neither did all professionals find orthogeriatric care professionally challenging.

## Background

For decades, hospitals have been organized vertically with each department functioning as an independent unit or “silo”, with the risk that specialization leads to fragmented and one-sided views of patient care and treatment [1]. Communication and coordination of patient care among the specialties have been found lacking [2]. Poor interprofessional collaboration may have negative effects on the delivery of health services and patient care [3]. Lately, initiatives such as the implementation of case management and integrated care pathways have proliferated in the attempt to diminish the gap between health specialties and professionals and to ensure quality in patient treatment and patient-perceived quality [4, 5].

The introduction of orthogeriatric care aims at improving quality in patient care for elderly patients admitted with fragility fractures. Treatment and care have traditionally been led and conducted by orthopaedic surgeons while consultation with geriatricians occurred only on an ad hoc basis. In contrast, orthogeriatric care is an interprofessional collaboration model in which geriatricians and orthopaedic surgeons work together supported by a team of nurses, nursing assistants, physiotherapists, occupational therapists and others [6].

While previous studies of orthogeriatric care models have found improved collaboration among health professionals, challenges have also been documented. An interview study involving 48 healthcare professionals evaluated interprofessional collaboration in discharge planning for patients admitted with a fractured neck of femur found little indication of enhanced interprofessional relationships and communication. In particular, the absence of goal-setting among interprofessional teams appeared to continue to challenge progress [7]. In another study, 16 clinical leaders from different disciplines participated in facilitated action meetings aiming at exploring collaborative approaches to the implementation of person-centred hip fracture care [8]. Christie et al. found that individuals, teams and management entertained essentially different expectations of goals and outcomes of the patient pathway. They also found that the professionals identified more strongly with their “own” group than with their interprofessional colleagues, thus retaining their individual professional identities. Overall, the findings indicate that 1) care continued to be delivered by distinct “service units”, 2) the professions worked independently of each other and 3) communication was insufficient; thus contributing to fragmented treatment and care. However, the introduction of facilitated action meetings was found to enhance communication by developing a patient-centred approach, shared values and overall understanding of the necessity of professionals’ different competences [8]. With Christie’s focus on the clinical leaders’ perception, the aim of our study was to describe clinical healthcare professionals’ views on and experiences with working in an interprofessional orthogeriatric unit. The study was conducted 2 years after the unit had been established.

## Method

We used focus groups to explore the healthcare professionals’ views on and experiences with working in an interprofessional orthogeriatric unit.

Our objective was to obtain data in a forum allowing the participants to deliberate on their own position in the context of the views of others, as recommended by Patton [9]. Focus groups are considered highly effective method for qualitative data collection for the exploration of attitudes and experiences among groups of people with potentially conflicting interests [10].

### Setting

The orthogeriatric unit for acute elderly patients admitted with fragility fractures was opened on 1 March, 2014, as a section of an orthopaedic surgery department at a regional hospital serving a mixed urban and rural district.

While staffing was essentially unchanged, the structures and processes guiding interprofessional collaboration were changed. Tasks were distributed in a new way and an agreement on shared responsibility for treatment signed by all staff.

The new structures and processes meant that nurses and nursing assistants were no longer dedicated to specific patient groups or categories. For the physiotherapists, their new schedules gave them several full days in the unit as opposed to earlier when they would visit several wards during the day. The single occupational therapist’s affiliation was extended to a half day weekly. The orthopaedic surgeons’ duties changed in that their brief and intermittent appearances on the ward were supplanted by regular attendance every morning for approximately 3 h, corresponding to the geriatricians’ presence. In the earlier regime, a single geriatrician would visit the department twice a week for 30 min to suggest medical treatment to be implemented by the orthopaedic surgeons.

Every weekday interprofessional team meetings were held in which all professions at work on the day were represented to secure shared goals and optimal treatment. The meetings usually lasted about 20 min. They were followed by meetings in smaller interprofessional groups for the coordination of patient care.

### Sampling and participants

Purposive convenience sampling was used for recruitment to the focus groups to ensure a varied and broad representation of perspectives on working in interprofessional teams [9]. Experience with the former organization was also taken into account. Because the nursing staff had experienced a high turnover after the implementation of the orthogeriatric unit, nurses who had left the ward were also invited to participate in the study.

Invitations were sent by email to 28 employees (a sample of approximately 50 healthcare professionals affiliated with the unit) – seven therapists, seven nurses (three of whom had left), two nursing assistants, four geriatricians and eight orthopaedic surgeons. Five were unable to participate and four (one physiotherapist, one geriatrician and two surgeons) did not respond to the invitation. Of the 19 participants recruited, 15 were women, four were men. Their ages varied between 27 and 63 years of age (mean 42.3 years); seniority at current place of work varied between two and 20 years (mean 7.9 years).

Four focus group interviews were conducted approximately 2 years after the implementation of the orthogeriatric unit. They took place at the hospital during day shifts. The composition of groups appears below:

- Focus group 1: Three physiotherapists and one occupational therapist (therapist group)
- Focus group 2: Four nurses and two nursing assistants (care group)
- Focus group 3: Three nurses (former employees)
- Focus group 4: Two geriatricians and four orthopaedic surgeons (physicians)

The focus group meetings were attended by the interviewer (first author CA) and a moderator (a clinical physiotherapist). Both were well known by all participants.

The 45–60-min long meetings were audio-recorded. Immediately after the interviews the moderator and the interviewer prepared notes. The interviews were transcribed verbatim. The interviewees were not invited to comment on the transcripts.

### Interview guide

The focus group interviews followed a thematic guide (Table 1) developed on the basis of health care professionals’ responses to a questionnaire on readiness for working in an orthogeriatric unit [11] and on CA’s observations in the unit.

**Table 1:** Thematic interview guide

*Research themes*	*Research questions*
Views on orthogeriatric care	How do you feel about working in the orthogeriatric unit? What is your attitude towards the establishment of the orthogeriatric unit?
Experiences with interprofessional collaboration in orthogeriatric care	What is your experience of orthogeriatric collaboration? What do you find important in interprofessional collaboration? What works well in the current interprofessional collaboration? What do you see as the possible challenges of interprofessional collaboration? Has collaboration with other professional groups emerged or has the collaboration attained a new meaning for your work?
Experiences with clinical aspects of orthogeriatric care	What has the new orthogeriatric organization mean for your clinical work? What work-related tasks/functions do you find particularly important in the clinical work? What do you see as the possible challenges in clinical orthogeriatric care?What do you see as the possible benefits of clinical orthogeriatric care?

As our ambition was to elucidate the participants’ attitudes as well as their experiences after the interprofessional orthogeriatric unit had been established for 2 years, our questions were rooted in an understanding of orthogeriatrics as both a clinical discipline and a collaboration model (Table 1).

The interview guide was prepared to ensure that all topics were covered; however the guide was not followed slavishly. Open-ended questions were used to encourage discussion; the participants’ viewpoints were validated by asking clarifying questions No other materials were utilised.

### Analysis

The focus group interviews were analysed using systematic text condensation (STC), as developed and described by Malterud [12]. A review of each interview was first conducted to form a general impression of the text and to identify preliminary themes. After re-reading the material, the texts were analysed according to meaning units, which were subsequently coded by first author. All authors collaborated on the reflection on findings and identification of themes. In the third step, each code group was condensed for further abstraction and final reconceptualization.

### Ethics

The participants received an invitation by email from the first author giving details and explaining the purpose of the focus group interview. They were further informed that the interview was confidential, that participation was voluntary and that they could withdraw at any time without consequences.

The processing of the collected data ensured that no individual could be identified. All quotations were ascribed to the professional affiliation of the source. Participation in a focus group was considered informed consent.

## Results

Across all four focus groups the participants agreed that the patients had benefitted from the introduction of orthogeriatric care and treatment. The reasons given were that all issues relevant for the patients’ condition and well-being were addressed, and that services were delivered in close collaboration among relevant healthcare professionals, community care and the family.

Three major themes emerged during the analysis: 1) A patient-centred approach, 2) An opportunity for professional growth and 3) Benefits of interprofessional collaboration. Below, the three themes are elucidated and illustrated by quotations.

### A patient-centred approach

#### How do professionals refer to the patient?

In the focus group discussions, the geriatricians and the care group in particular focused on the patients, who were frequently referred to and mentioned as the key element in their work and routines.

Why are we here? Is the point that I can perform my nursing job – or do things start with the patient (…) I think it starts with the patient (…). (Nurse 6)

In contrast, the therapists and the orthopaedic surgeons rarely mentioned the patients and merely did so in relation to the services provided.

As an orthopedic surgeon, we believe that the hip fracture is the smallest problem (…) (Orthopaedic Surgeon 1) The care group stressed the fact that patient pathways had become individualized and that the focus on patients’ various needs and problems had increased. This had meant that nurses now saw the individual patient as someone with varying and individual needs rather than, for example, “a knee-replacement patient”.

[W]e take care of the whole patient, not just a small part (…). (Nurse 6)

The new collaboration model thus seemed to encourage the nurses to take a more holistic and patient-centred approach.

A similar approach was expressed by the geriatricians. They referred to the patients as individuals, rather than just a fracture. In fact, one used imagery to describe her sense of expectation.

Each patient is like a riddle, like a gift you want to open and uncover. [Let’s find out] what their real problem is, so we can find the best solution (…). (Geriatrician 1)

By referring to “the real problem”, the geriatricians pointed to the underlying condition causing the fracture. In their view, if the root problem was not addressed, patients were merely treated symptomatically, or “fracture by fracture”. The holistic and patient-centred approach furthermore appeared from their emphasis on the importance of meeting the patients’ post-operative challenges and from their consideration of the patient’s domestic conditions and social network.

#### Shared views of the patient

The care group acknowledged the geriatricians’ view of the patient, which they contrasted with that of the orthopaedic surgeons.

Compared to the orthopaedic surgeons, [the geriatricians] see and hear the patient in a whole new way. (Nurse 1)

This is seen as a token of the shared views and approaches among the care group and the geriatricians.

Some of the interviewed orthopaedic surgeons referred to the patients in a more narrow sense in that they focused mainly on specific surgical procedures and the fracture that had led to admission. During the interviews, it became evident that some orthopaedic surgeons continued to focus on the fracture and to a lesser degree on the person with a fracture.

[The patients] are orthopedically fully treated when the operation is over (…) (Orthopaedic surgeon 3)

However, some of the orthopaedic surgeons appeared to have adopted a broader view of the patient.

Taken broadly, the therapists’ approach seemed to reflect that of the orthopaedic surgeons. Some therapists appeared to view their patients through the optics of fracture-relevant training exercises and their potential for full rehabilitation. They primarily focused on the fracture and training tasks rather than on the patients’ needs and wishes.

We solve the hip-related problems (…) and we will assess, plan and train [the patients] as well as possible (…) (Therapist 3)

However, some therapist also recognized and spoke about the patients in ways that reflected the complexity and variety of needs.

In general, the groups agreed that the introduction of orthogeriatric care had enhanced the quality of treatment.

### An opportunity for professional growth

The analysis identified the perception of opportunities for professional growth as a recurring theme. The different professions’ views on their work in the orthogeriatric unit varied to some extent.

Some of the nurses indicated that the merger of the two specialties had stimulated their professional development and enhanced the opportunity for professional growth by training in different and more complex medical issues, learning to react on acute medical conditions and working with patients with complex needs.

I find it challenging – professionally as well as personally. There are lots of problems to attend every day (…) It’s a challenging patients group (…) you constantly learn new things. (Nurse 4)

Orthogeriatric care requires complex clinical observation and evaluation of patients and thus called for professional skills development. The same nurses found that working in the orthogeriatric unit had given them back their job satisfaction. Their professional pride was clearly evident.

The geriatricians considered their skills and professional contribution highly relevant for the orthogeriatric patients.

[I]t really makes sense that when we admit people who have fallen, we start by looking for reasons why it happened – and that reason is often medical (…) so our contribution is clearly relevant (…). (Geriatrician 1)

As orthogeriatric patients typically have multiple comorbidities requiring polypharmacy and are at high risk for developing postoperative medical complications, the geriatricians found their competences fully exploited.

Among the orthopaedic surgeons some voiced concern that their professionalism was challenged by the unit’s strongly medical focus. In terms of surgical skills, they said their competences were used appropriately.

In contrast, the therapists indicated that working only with orthogeriatric patients posed little challenge to their professionalism. Despite the diversity of fractures and patients, the daily repertoire of training exercises was very restricted. They found that the training of orthogeriatric patients did not always deliver sustainable results.

The work is very monotonous (…). I’d get bored with that group of patients if I had to work there every day. (Therapist 1)

The factors mentioned above led some of the therapists to give voice to a degree of demotivation, boredom and lack of professional growth. In their view an organization with few dedicated therapists working full-time in the orthogeriatric unit was not conducive to professional development. The group missed opportunities for professional discussion and feedback on the choice of exercises, motivational work, and indication for specific treatments to complex patients.

#### Professional challenges

However, the focus group interviews also exposed conflicts and disagreement. The majority of nurses and nursing assistants found their job very busy and stressful. Their many tasks included a range of care acts, clinical testing and medication as well as communication with families and municipal home care units, all of which they saw as relevant and essential. Yet, some experienced work overloads that forced them to prioritize among their duties, with the result that some tasks tended to be ignored.

[T]hinking back, there are many days you didn’t manage to do all the things you wanted to do (…). (Nurse 4)

As a consequence, rather than care and treatment being guided by clinical measurements and tests results, they became unfinished business on busy days. Some nurses and nursing assistants felt inadequate; when their task prioritization or omissions were occasionally commented by colleagues, they felt like firefighters without enough water in the tank.

Some of the nurses who no longer worked in the unit mentioned the heavy workload and the feeling of inadequacy as reasons for leaving. Moreover, nurses with long-term employment in the department had viewed the reorganization as a welcome opportunity to seek new challenges. A snowball effect was also mentioned; the nurses had all experienced the catalysing effect of resignations, especially during periods with heavy workloads or constant change in the department.

Although in general the orthopaedic surgeons recognized the need to offer medical treatment to elderly patients with fragility fractures, they mainly considered their responsibilities to be restricted to the fracture. In particular, the surgeons seemed challenged by expectations from the care group that they offer assessments of geriatric and acute test results and initiating treatment in situations where a geriatrician was unavailable. The orthopaedic surgeons admitted to occasional feelings of insufficiency and incompetence.

Although some of the therapists enjoyed working with orthogeriatric patients, the majority considered the elderly as “a heavy workload” in that they required much practical help and motivation before and during training sessions.

[I]t is physically and mentally challenging to handle patients you can’t always communicate with (…). (Therapist 3)

The therapists found that the cognitive impairment of some of the patients hindered communication, while others were too weak or unwell. They were challenged by the dilemma posed by their professional focus on rehabilitating patients to the highest possible level and their patients’ poor motivation or capability. In the group, the scarcity of time and the many tasks had occasionally created disagreement over the prioritization of patients. Moreover, collaboration with nurses and nursing assistants on patients’ rehabilitation potential likewise tended to be strained as the carers typically expected more intensive training than the therapists saw as feasible.

### Benefits of interprofessional collaboration

In all focus groups, it was mentioned that interprofessional collaboration had increased considerably after the orthogeriatric unit was established. In particular the systematic and frequent face-to-face communication at the interprofessional team meetings was emphasized as essential to the sense of collegial solidarity and interprofessional collaboration.

The therapists elaborated on the significance of taking an equal and active role in a larger team with responsibility for treatment. Their sense that their colleagues in the unit expected them to participate in meetings and valued their information on optimal training positions and goals had made them feel more respected and integral parts of the team.

[T]hey listen more and they know they can count on us (…) (Therapist 1)

[W]hen we see [the patients], they’ve had surgery (…), then there’re medical issues and then we come in to train ADL [activities of daily living] and physiotherapy – everything carries equal weight (…). (Therapist 3)

The therapists found the new procedures with face-to-face requests had stimulated their sense of ownership and flexibility. The earlier routine with written request had made it easy to treat things strictly by the rules, to postpone or even avoid.

[Before, you] would come [to the unit] for patients signed up for training – you would have those three [patients] to see, and then you’d be gone. (Therapist 3)

Furthermore, the care group and the therapist group found that meeting each other every day had improved interprofessional relations and made it easier to ask for advice or lending a helping hand.

[N]ow you know who to contact. Having informal contacts makes things much easier (…). That’s the benefit of working on the same ward. (Therapist 4)

#### Different experiences of collaboration

The focus group discussions of interprofessional collaboration highlighted differences in expectations and experiences. The care group indicated that they saw good communication, mutual respect, acknowledgement and a shared focus as valuable elements in the interprofessional collaboration. They spoke very positively about their collaboration with the physicians and the therapists, which they found was characterized by respect and a strong teamwork guided by clear goals. However, as in the nurses’ view, the therapists had a weak position in the team, they discussed how to invite the therapists to get more involved in the collaboration, especially in the team meetings.

I think that nurses and physicians do most of the talking at team meeting and we could (…) do more to encourage the therapists (…). (Nurse 6)

The physicians’ assessment of the teamwork showed that they valued collaboration and were respectful of the knowledge, competences and input offered by their interprofessional colleagues.

“[E]veryone provides input – when the physiotherapists report that the patient can’t walk because of their constant pain, the orthopaedic surgeon needs to take care of that (…) or the nurses say that the patient is not eating properly (…).” (Geriatrician 2)

In particular, the geriatricians and the surgeons appreciated the advantages of access to consultation with each other in complicated cases.

The geriatricians emphasized the value of good communication and everyone working towards a shared goal; illustrated by a dogsled.

[A] physiotherapist, a doctor and a nurse or two are buckled up in front of a dog sledge carrying a gift [the patient] – we’re all pulling in the same direction. We can all bark about the good things we see and hear – that way, it’s easier to reach the same destination. (Geriatrician 1).

*Drawing by Geriatrician 1.*

The dog sledge symbolizes a focused, efficient and coherent care pathway in which all healthcare professionals pull together towards the same goal. If someone takes another direction or does not contribute his fair share, the sledge will slow up or start wobbling, with the result that the care pathway is challenged and possibly retarded.

However, not all orthopaedic surgeons saw the relevance of all the information from their collaborators, which they found unnecessary for their decisions on the appropriate surgical treatment.

It’s not relevant for a surgeon to know whether home care is ready or not, or whether a bed has been requested or walking aids are in place (…). (Orthopaedic surgeon 3)

When their opinion on interprofessional collaboration was elicited, the therapists emphasized the value of continuous communication, a respectful attitude, a readiness for listening to one another, and improved knowledge of the other professionals’ tasks. While acknowledging the importance of interprofessional collaboration, they considered it as practically restricted to the interprofessional team meetings. The therapists also indicated that their benefit from the meetings were minimal as they deemed some of the information irrelevant for them. Although collaboration with the care group was viewed as positive, some therapists found that they were met with unrealistic expectations about the time and tasks that therapists could dedicate to the unit. They were placed in a dilemma arising from the poor match of expectations from, on the one hand, their interprofessional colleagues and, on the other hand, their professional colleagues and manager.

These reservations notwithstanding, all therapists recognized the great advantage of a concerted effort on treatment and found that task coordination and continuity had improved.

## Discussion

All the professionals interviewed for our study agreed that the reorganization of orthogeriatric care had improved quality in the treatment of elderly patients admitted with fragility fractures as the new organization addresses all relevant issues for the patients’ condition and well-being. The reorganization furthermore supported the healthcare professionals’ interprofessional collaboration towards common goals; as a staff member illustrated by drawing a dogsled with everyone pulling in the same direction.

Collaboration among the professions appears to have been strongly stimulated by the introduction of the orthogeriatric unit. In particular, the frequent face-to-face communication enabled by the new structure was considered essential for the increased sense of collegial solidarity and respect. Corresponding with the findings of Christie et al., we found that the daily meetings with the purpose of improving the patient journey and creating shared understandings and goals enhanced interprofessional collaboration. Whereas Christie et al. introduced a participatory process by inviting the healthcare professionals to participate in meetings over a limited period of time outside work settings [8], in the unit under study here the process aimed to establish a collaborative framework directly connected with patient care and treatment. It appears that whereas frequent meetings can improve interprofessional collaboration and the patient care pathway, in themselves they do not ensure improvement if shared goal-setting is absent [7].

A number of other positive elements in the changes reflected the sense of community as illustrated for example by the therapists’ experience that their increased presence had led to better appreciation of their work and acceptance as team members. Other examples are the nurses’ greater experience of shared responsibility and the physicians’ experience of followership.

However, the interprofessional collaboration continued to be challenged 2 years on. Conflicting expectations appear to be inherent in interprofessional collaboration, as exemplified by the therapists, who experienced cross-pressure in balancing obligations in the orthogeriatric unit and the therapy department, or by surgeons experiencing an increased pressure to respond to medical questions.

The greatest challenge appeared to concern professional satisfaction and growth. Whereas some of the healthcare professionals experienced great satisfaction and even professional growth, others gained little professional gratification from treating and caring for their medically complex and frail patients. This may be explained by differences in the professional groups’ socialization and education. Therapists and physicians are trained to focus on performance, outcomes and improving the patient’s condition, whereas nurses are trained with a view to improve the patient’s quality of life by providing good care. [13]. Therapists and surgeons in particular appeared to find it challenging to treat elderly patients with complex problems and seemingly little potential for full rehabilitation. In contrast, a geriatrician who has chosen to specialize in frail elderly patients appears to be more likely to find job satisfaction in an orthogeriatric unit. In the case of the nurses, our findings suggest that despite having trained for the care of patients and their quality of life, individual interests and workload seem important.

Even though interprofessional collaboration has improved over the course of the 2 years since the orthogeriatric unit was established, views and expectations among staff continue to be embedded in professional interests and organizational cultures, and changes thus occur very gradually.

### Strengths and weaknesses

Our data on interprofessional collaboration were collected in uniprofessional focus groups, which enabled the participants to express their views without being influenced by outsiders’ views and interests. In interprofessional fora including physicians, it has been found that therapists and nurses restrain themselves in voicing their opinion [14]. With focus groups representing several professions, we may have been able to better reveal the complexities of interprofessional collaboration. While our sampling of participants aimed at securing the greatest possible variation in perspectives, it was also guided by the duty roster so that the participants were inconvenienced as little as possible.

The interviewer (CA) was well known to many of the informants but no professional affiliation existed. As the assessment of orthogeriatric care was requested by the Department of Orthopaedic Surgery, informants may have expressed a more positive experience than would otherwise be the case.

To ensure the validity and reliability of the analysis, we varied our questioning technique. The coding was performed as an iterative process in collaboration among all authors.

Our findings are based exclusively on the views expressed by the 19 focus group participants; hence, we are not aware of the views and experiences of other professionals working in the orthogeriatric unit.

## Conclusion

The introduction of orthogeriatric care was seen to have improved the quality of treatment by all professionals. However, work routines were challenged by heavy workloads that in some cases manifested in stress symptoms. Furthermore, the enhanced communication, broader appreciation of competences and the sense of a shared goal were seen to have resulted in interprofessional collaboration. Nevertheless, 2 years after its implementation, the orthogeriatric model continued to be challenged by different expectations among the various professions. Neither did all professionals find orthogeriatric care sufficiently stimulating.

## Abbreviations

ADL: Activity of daily living
STC: Systematic text condensation
